# Single-molecule techniques to visualize and to characterize liquid-liquid phase separation and phase transition: Single-molecule techniques to visualize LLPS and transition

**DOI:** 10.3724/abbs.2023028

**Published:** 2023-03-03

**Authors:** Jinyao Ji, Wenjuan Wang, Chunlai Chen

**Affiliations:** 1 School of Life Sciences Beijing Advanced Innovation Center for Structural Biology Beijing Frontier Research Center of Biological Structure Tsinghua University Beijing 100084 China; 2 School of Life Sciences Technology Center for Protein Sciences Tsinghua University Beijing 100084 China

**Keywords:** LLPS, phase transition, single-molecule techniques

## Abstract

Biomolecules forming membraneless structures via liquid-liquid phase separation (LLPS) is a common event in living cells. Some liquid-like condensates can convert into solid-like aggregations, and such a phase transition process is related to some neurodegenerative diseases. Liquid-like condensates and solid-like aggregations usually exhibit distinctive fluidity and are commonly distinguished via their morphology and dynamic properties identified through ensemble methods. Emerging single-molecule techniques are a group of highly sensitive techniques, which can offer further mechanistic insights into LLPS and phase transition at the molecular level. Here, we summarize the working principles of several commonly used single-molecule techniques and demonstrate their unique power in manipulating LLPS, examining mechanical properties at the nanoscale, and monitoring dynamic and thermodynamic properties at the molecular level. Thus, single-molecule techniques are unique tools to characterize LLPS and liquid-to-solid phase transition under close-to-physiological conditions.

## Introduction

A decade ago, researchers observed P granules in *Caenorhabditis elegans* and nucleoli in *Xenopus* oocytes both exhibiting liquid-like features, bringing liquid-liquid phase separation (LLPS) into the field of biology [ 1, 2] . Biomolecules undergoing LLPS are usually driven by multivalent weak interactions among molecules to form reversible membraneless structures, which concentrate specific components into the condensed phase to ensure or facilitate the correct biological processes in the complex intracellular environment [ 3, 4] . Intriguingly, a group of neurodegenerative-related proteins are reported to be able to self-assemble into reversible liquid-like condensates and to gradually mature into solid-like aggregations with fibrous structures over time [ 5– 10] . Moreover, these aggregations resemble the disease-associated inclusions [ 11– 15] . Several disease-related mutants have been identified to accelerate liquid-to-solid conversion [ 16– 20] . Hence, a close investigation of LLPS and phase transition would contribute to a deeper understanding of the liquid-to-solid transition and possibly the mechanisms underlying related diseases.

Liquid-like condensates and solid-like aggregations exhibit distinctive properties. The surface tension keeps liquid-like condensates spherical, whereas gel-like intermediates and solid-like aggregations can adopt irregular shapes. Fluorescence microscopy is the most commonly used technique to identify different morphologies and to distinguish liquid-like condensates from solid-like aggregations [16]. Due to the optical diffraction limit, only structures at the micrometer scale or larger can be visualized by commonly used fluorescence microscopy. Super-resolution fluorescence microscopy techniques, such as simulated emission depletion (STED) microscopy and stochastic optical reconstruction microscopy (STORM), are needed to reveal structures at tens and hundreds of nanometers [ 19, 21] . Electron microscopy is also able to visualize nano-structures of solid aggregations [ 9, 10, 22, 23] . Besides morphological features, condensates are more dynamic than aggregations. Small condensates can fuse into large condensates, and molecules within condensates can diffuse and exchange with the surroundings quickly, whereas solid-like aggregations cannot. Fluorescence recovery after photobleaching (FRAP) is a benchmark to examine fluidity [ 16, 20, 24– 26] . Specifically, the fluorescence of laser-bleached regions within droplets can quickly recover due to the rapid exchange of molecules between droplets and their surroundings, whereas recovery of solid-like aggregations is slow or even negligible. Besides the translational diffusion examined by FRAP, fluorescence anisotropy measurements can capture the rotational dynamics of molecules on the picosecond to nanosecond timescales [27]. In addition, 1,6-hexanediol is used to disrupt weak hydrophobic interactions to dissolve or suppress condensates formed by reversible weak interactions, whereas irreversibly matured aggregations are barely affected by 1,6-hexanediol [ 25, 28, 29] . These ensemble approaches are well established and widely used to study the properties of condensates and aggregations.

Single-molecule techniques are highly sensitive and can examine or even manipulate individual molecules or particles, providing unique quantitative information that cannot be obtained by ensemble methods, such as morphologies, mechanical properties, and condensate sizes at the nanoscale or even at the atomic resolution, dynamics of intramolecular conformations and intermolecular interactions ranging from nanosecond to minute, molecular diffusion coefficients and binding affinities in the condensed and diluted phases, and so on. Various single-molecule techniques have been used to examine liquid-like condensates and solid-like aggregations, revealing information complementary to the commonly used techniques mentioned above and gaining further mechanistic insights to understand LLPS and phase transition. Herein, we summarize the basic principles of several single-molecule techniques and demonstrate their power with example cases.

## Applications of Various Single-Molecule Techniques in LLPS and Phase Transition

### Atomic force microscopy

**Figure 1:**
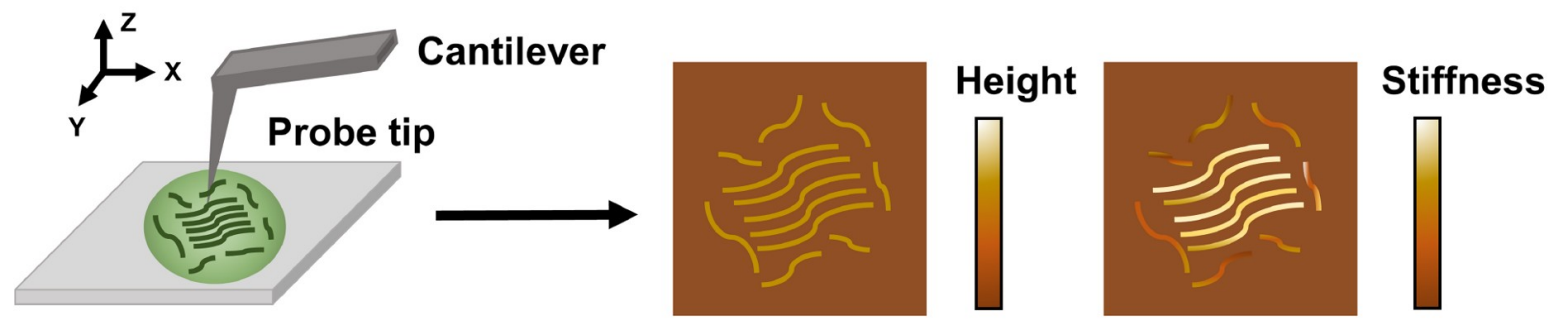
Figure 1 The schematic diagram of AFM AFM obtains the surface morphology and mechanical properties via scanning the condensate surface with the cantilever and the probe tip.

Atomic force microscopy (AFM) consists of a micro-cantilever with a sharp probe tip, a control, and a feedback device to sense the weak repulsive or attractive force between the sample surface and the probe tip. During measurements, the cantilever is scanned on the sample surface, and its position and sensing force are recorded ( Figure 1). AFM can operate in contact mode, non-contact mode, and tapping mode to obtain the surface morphology up to atomic resolution and other mechanical properties, such as stiffness, adhesion, and Young’s modulus [30]. The advantage of AFM is that it can work under close-to-physiological buffer conditions to examine biomolecules, macro-complexes, and cells, whose sizes range from the nanoscale to the micrometer scale.

AFM is a powerful tool to acquire the morphology, mechanical property, and heterogeneity of complexes at the nanoscale. Hydrogels and aggregations containing fibrous structures formed by TDP-43 and tau at the nanoscale have been visualized without the need for specific fixation [ 31– 33] . Besides the morphology, condensates formed by asymmetrically dimethylated fuse in sarcoma (FUS) and hypomethylated FUS, which possess liquid- and solid-like properties, respectively, display distinctive intrinsic stiffness straightforwardly in AFM detection [34]. In addition, AFM can be combined with other spectroscopy methods to provide enriched information. For example, AFM-based infrared nanospectroscopy can correlate the nanoscale stiffness of FUS condensates with their secondary and quaternary structural properties to provide further molecular mechanistic insights regarding how stable condensates are formed [34]. The direct correlation of mechanical and structural information further broadens the applications of AFM to visualize regional heterogeneity during the liquid-to-solid transition. Moreover, AFM can capture condensate growth and fibril formation in real time. Studies have shown that mRNA serves as the scaffold for the aggregation of TIA-1, TDP-43, and FUS, which can be dissociated upon the addition of the stress granule proteins YB-1 and G3BP1 [ 35, 36] . Similarly, the recruitment of FUS to DNA damage sites has been examined using an *in vitro* reconstituted LLPS system [37]. Time-lapse AFM imaging reveals that amlyin fibrils grown in the test tube and on the mica surface exhibit distinct features [38]. Via high-speed AFM detection, fibrils formed by amyloid β-protein (Aβ) have been found to have two growth modes and can switch from one growth mode to another, producing either straight or spiral fibrils [39]. Similarly, the formation of β-lactoglobulin fibrils has been examined by AFM to propose a general model for amyloid fibril assembly [40], which would inspire the applications of AFM in LLPS-involved fibrillar systems.

Without the need for the harsh sample preparation used for conventional electron microscopy imaging, which might disrupt the complexes, AFM is a powerful tool to visualize samples in their native state in real time. The ability of AFM to directly acquire the morphology, mechanical property, and growth process of condensates at the nanoscale shows great potential in revealing the structural information of condensates.

### Optical tweezers

Optical tweezers, also known as optical traps, use highly focused laser beams to produce small optical forces on the order of piconewton to hold and manipulate particles and objects in all three dimensions [41]. Multiple laser beams can be used to create several optical traps to manipulate multi-particles at the same time. In addition, optical tweezers equipped with optical microscopes and fluorescence microscopes can capture the dynamic motions of the trapped particles and labeled molecules of interest simultaneously. Optical tweezers are rarely used to directly trap individual molecules. Instead, biomolecules are anchored between two or more laser-trapped beads for further manipulation and observation.

**Figure 2:**
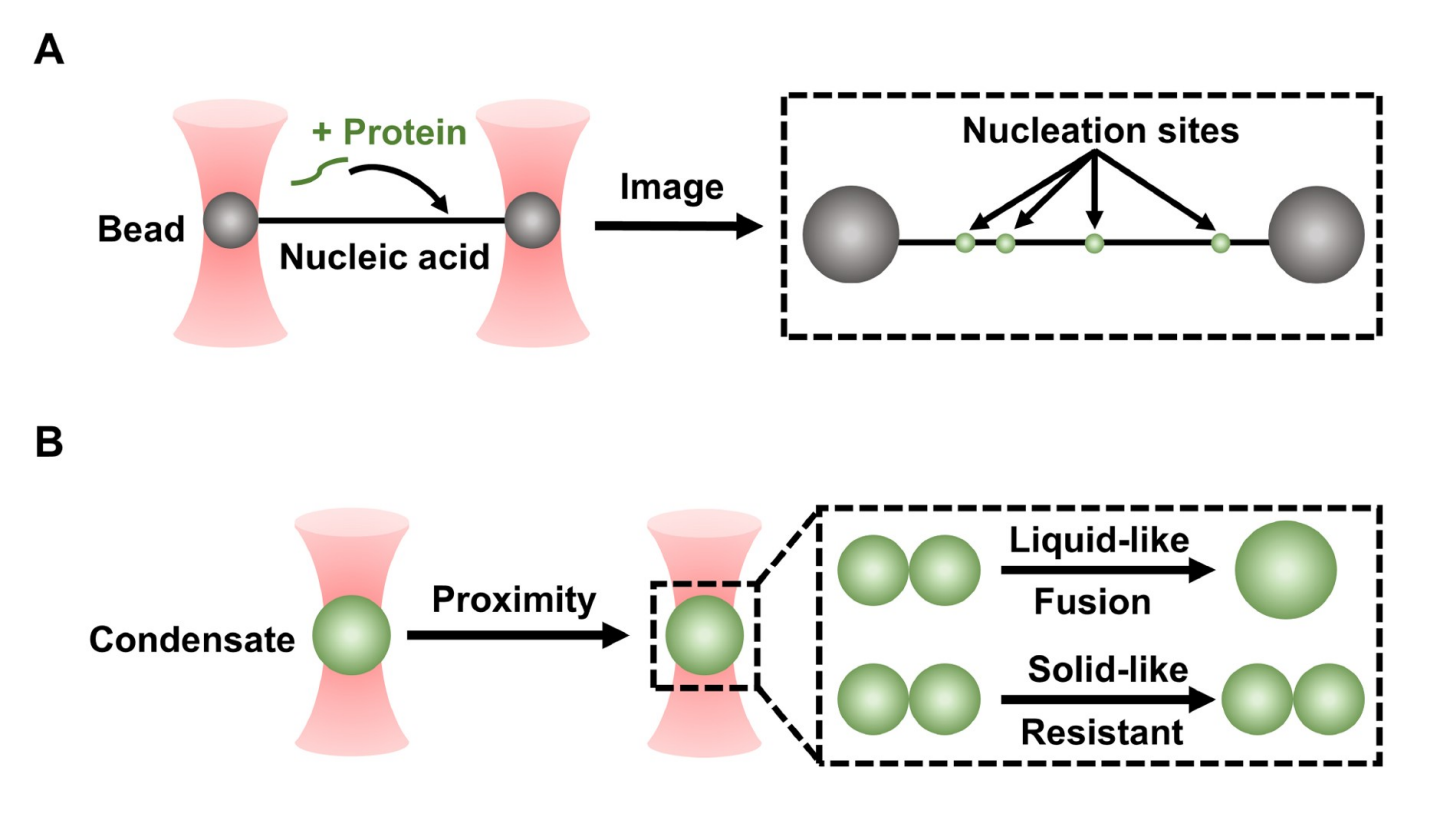
Figure 2 The schematic diagram of optical tweezers Optical tweezers can (A) reveal the nucleation sites on the single nucleic acid strands and (B) characterize the fused fraction among total condensates and their fusion time.

Optical tweezers have been used to quantitatively characterize the nucleation and fusion of condensates. Using stretched double-stranded DNA (dsDNA) between two trapped beads ( Figure 2A), FUS has been found to form a single layer on dsDNA, which is an important nucleation mechanism for condensation [42]. FUS-DDIT3 condensates on trapped dsDNA can further recruit BRG1, the core subunit of SWI/SNF, to alter chromatin dynamics and rewire transcriptional programs [43]. With a similar experimental design, the human H1.4 protein is found to coalesce around nascent single-stranded DNA (ssDNA) under no tension, which can further form phase-separated condensates [44]. Combining optical tweezers and theoretical analysis, another study demonstrated that the transcription factor Klf4 adopts a concentration-mediated switch-like transition from an absorbed state to a condensed state in a sequence-dependent manner when binding to DNA, which follows a heterogeneous Ising model [45]. Hence, optical tweezers provide a general workflow to visualize the condensation pattern between nucleic acids and proteins and to examine the functions of phase separation to regulate biological activities via further biomolecule recruitment.

Due to different refractive indices relative to the diluted phase, the condensates can be directly captured by a dual-trap system and brought into proximity to quantify their fusion behaviors ( Figure 2B). Condensates with fluid properties can fuse into one droplet quickly, while those that possess solid-like properties will take much longer time to fuse or resist full coalescence [ 44, 46, 47] . An interesting study revealed that the fusion of two micrometer-sized hollow nucleoprotein-RNA condensates quickly reforms a new hollow condensate with a single compartment, indicating the formation of vesicle-like ordered assemblies [48]. Optical tweezer-controlled fusion is an effective and quantitative indicator to characterize whether a condensate is fully or partially liquid-like or solid-like even though its shape remains spherical.

### DNA curtains

DNA curtains are novel high-throughput assays to visualize protein-nucleic acid interactions in real time, which combine nanofabricated surface structure, lipid bilayer-coated glass slide, and hydrodynamic force to align hundreds or even thousands of DNA molecules into designed patterns in a flow cell for single-molecule fluorescence measurements [49]. The lipid bilayer-coated glass slide is used for surface passivation and DNA immobilization. Based on specific experimental requirements, single-tethered curtains are constructed by anchoring only one end of long ssDNA or dsDNA strands, which are stretched in the flow chamber by continuous buffer flow, to the lipid bilayer, whereas double-tethered curtains are constructed by anchoring both ends of DNAs across nanostructures in the chamber without the need for continuous buffer flow. Usually, fluorophore-labeled proteins are loaded into the flow cell, and interactions between proteins and nucleic acids can be imaged by fluorescence microscopy.

**Figure 3:**
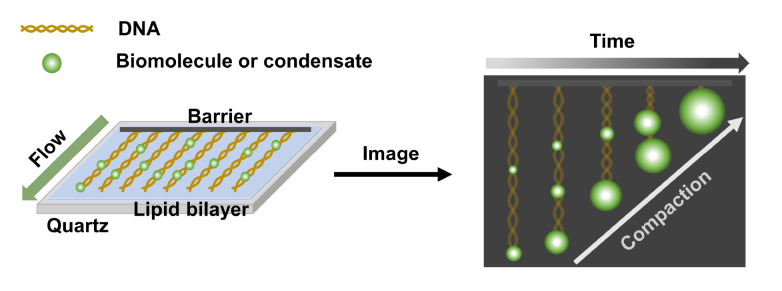
Figure 3 The schematic diagram of DNA curtains Single-tethered DNA curtains achieve real-time high-throughput visualization of protein compaction on DNA.

Shrinkage of DNA length and compaction rate are two major parameters quantified by DNA curtains to indicate the phase separation abilities of different proteins with DNA ( Figure 3). For instance, DNA curtains have been applied to visualize how HP1α and VRN1 compact into condensates on DNA molecules, offering direct access to spatiotemporal information at the millisecond timescale [ 50‒ 52] . According to the results of DNA curtains, the action of wild-type HP1α to DNA is cooperative with an initial appearance of single puncta followed by rapid compaction, while N-terminal phosphorylated HP1α exhibits multiple puncta with a slower compaction rate, indicating the cooperative binding to DNA is disturbed by phosphorylation [51]. Moreover, owing to the high-throughput nature of DNA curtains, fluorescence imaging enables researchers to directly and quantitatively identify the modulatory roles of DNA in LLPS. In a study conducted on FET fusion oncoproteins, DNA curtains revealed the preferred binding and nucleation sites, the threshold number of DNA elements to enhance condensation formation, and the transcription activity enhanced by the recruitment of Pol II CTD to the FUS-Gal4 condensates, none of which can be efficiently unraveled by traditional ensemble methods [53].

The participation of DNA regulates the formation and fluidity of condensates in many LLPS and phase transition systems. Therefore, it is important to clarify the role of DNA. Compared to optical tweezers, which can trap one or several DNA molecules for manipulation, DNA curtains are high-throughput detection methods that cannot detect forces. The direct real-time imaging of protein recruitment, DNA compaction, and condensate formation by a single DNA strand or by adjacent DNA molecules makes DNA curtains a unique platform to examine DNA-involved condensates.

### Single particle tracking

**Figure 4:**
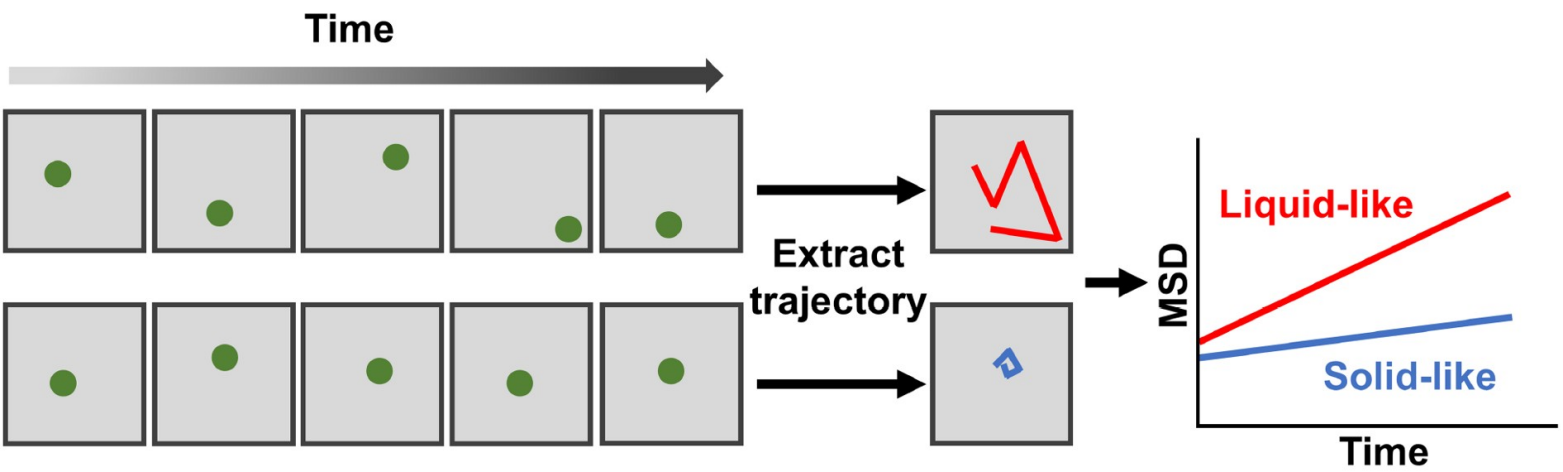
Figure 4 The schematic diagram of SPT SPT tracks the movement trajectories of individual molecules or particles to quantify their diffusion behaviors.

Single particle tracking (SPT) is a technology for monitoring the movement of individual molecules or particles with nanoscale precision. Normally, fluorescence signals of molecules or particles of interest are recorded in a consecutive time period, from which their locations are extracted to reconstitute movement trajectories over time for further analysis. The mean square displacement (MSD) calculated from fixed time intervals is a major parameter conveyed by SPT to quantify the diffusion behavior of the target molecules or particles or the viscosity of their surrounding environments ( Figure 4) [54].

In *in vitro* reconstituted systems, SPT has been applied to quantify the inner viscosities of FUS and protein-nucleic acid condensates by tracking the Brownian motions of fluorescent beads within condensates [ 12, 55] . The MSD of fluorescent beads within elastin condensates decreases significantly over time, indicating the liquid-to-solid transition [56]. SPT further reveals that the structural properties of guest molecules determine their diffusion dynamics within FUS condensates [57]. Besides fluorescence microscopy, dark-field microscopy has been used to simultaneously track the diffusion dynamics of multiple gold nanorods within protein condensates, revealing the spatiotemporal heterogeneity of phase separation [58]. By recording the diffusion behaviors of probes inside condensates, SPT characterizes the fluidity of condensates, which is helpful to monitor the liquid-to-solid transition and to map the heterogeneity of condensates.

SPT is also capable of tracking the movements of individual molecules within living cells. The spatial and temporal localization and regulation of mRNAs under stress conditions are examined, showing the translation and degradation rates of mRNAs within stress granules and processing bodies [59]. Within the nucleoid volume, the ParB protein exhibits two distinct dynamic behaviors reflected by low mobility and high mobility, corresponding to molecules in the diluted phase and those trapped inside condensates, respectively. In addition, ParB molecules are shown to rapidly diffuse between different condensates [60]. Chromobox protein 2 nucleates on chromatin for condensate assembly to facilitate its target search process by reducing diffusion time and visiting target sites repetitively [61]. For stationary particles displaying no significant movement, SPT can extract their residence times from appearance to disappearance. Residence time quantified by SPT is a key parameter to indicate that LLPS of low complexity domains facilitates the binding of transcription factors and gene activation [26]. Furthermore, the combination of FRAP and SPT permits reliable minutes-long FRAP analysis, diminishing artifacts caused by non-uniform backgrounds [62].

Although FRAP and SPT both characterize the fluidity of condensates, SPT can directly track the motions of individual molecules and particles *in vitro* and in living cells, which provides insights at the molecular level to understand how LLPS and phase transition affect the movements and interactions of molecules, hence modulating biological reactions and functions.

### Single-molecule fluorescence methods

**Figure 5:**
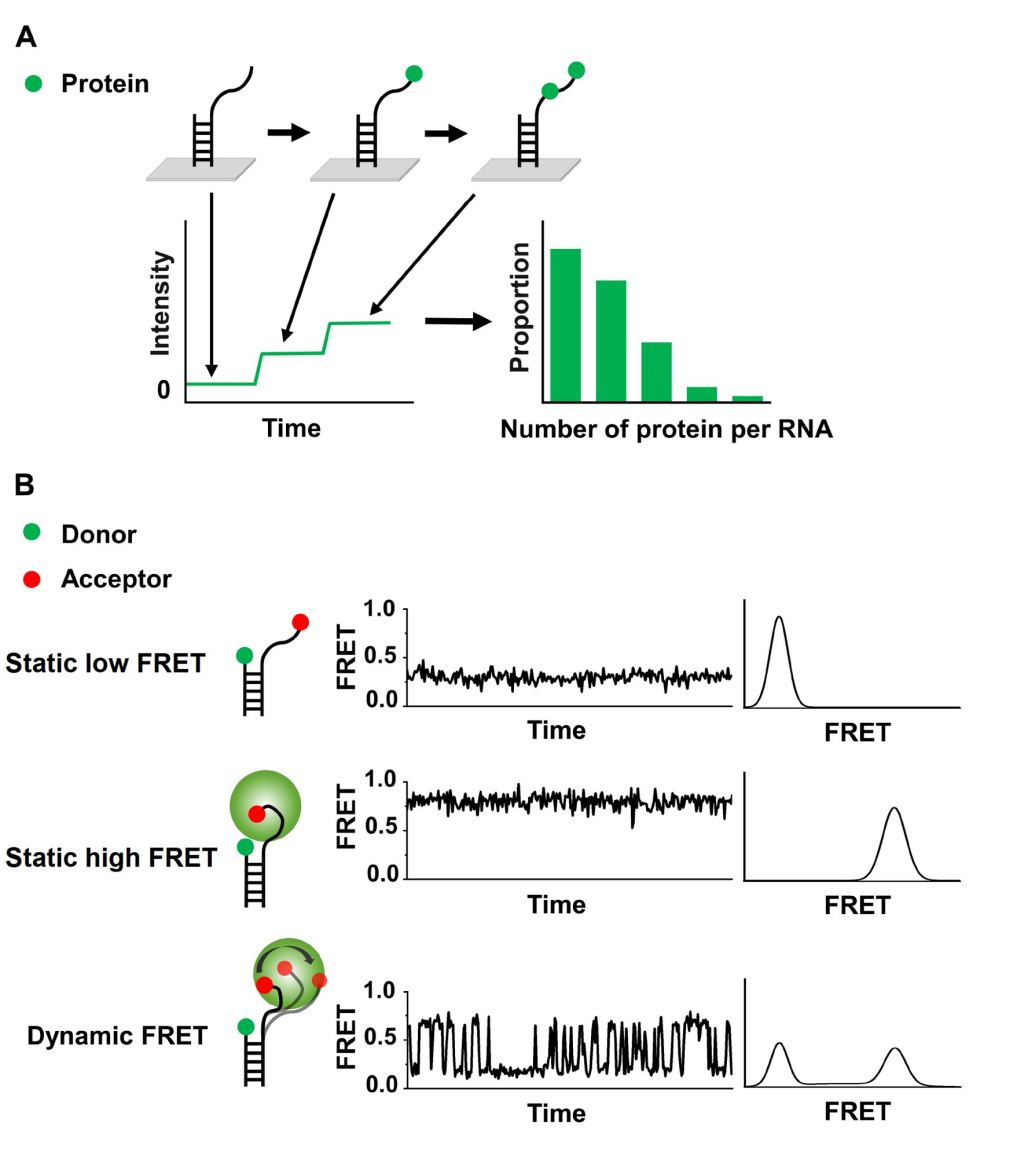
Figure 5 The schematic diagram of single-molecule fluorescence methods (A) Single-molecule counting captures the step-by-step increase in fluorescence signals caused by the binding of individual molecules. (B) Conformational dynamics of molecules affected by LLPS or their binding partners revealed by smFRET.

Using highly-sensitive photon detectors, single-molecule fluorescence methods capture fluorescence signals from individual biomolecules to characterize intramolecular conformational dynamics and intermolecular interactions. Using fluorophore-labeled FUS proteins, single-molecule counting reveals that each RNA molecule on average accommodates more arginine mutants than WT proteins, supporting that arginine mutants form larger condensates ( Figure 5A) [63]. In addition, researchers observed that WT and glycine mutants exclude each other from engaging the same RNA molecule, suggesting that WT and glycine mutants exhibit distinctive conformations and explaining why FUS mutations in glycine do not associate with WT FUS. A study from the same group showed that poly (ADP-ribose) mainly interacts with FUS via transient interactions, which is still sufficient to induce LLPS [64]. The finding demonstrated by single-molecule counting hints that the condensates formed by different mutants are distinct at the early stage of FUS-RNA nucleation, although these condensates display similar morphologies and liquid-to-solid phase transition behaviors under fluorescence microscopes. This case provides a new avenue to study the early stage of FUS condensates and the liquid-to-solid transition of other LLPS systems.

Single-molecule fluorescence resonance energy transfer (smFRET), also known as single-molecule Förster resonance energy transfer, is one of the most extensively used single-molecule methods. FRET is a distance-dependent photo-physical process during which energy is transferred nonradiatively from an excited donor fluorophore to an acceptor fluorophore [65]. The FRET transfer efficiency highly depends on the relative distance between donor and acceptor fluorophores. Thus, intramolecular conformational dynamics and intermolecular interactions, causing the change in distance between two labeling sites, can be captured by smFRET in real time ( Figure 5B).

To date, smFRET is mainly used to examine how intramolecular conformational dynamics of RNA and proteins are affected by LLPS or by their binding partners in LLPS. The dynamic information is hard to access using other structural biological approaches due to the intrinsically disordered nature of proteins and the liquid-like property of condensates. Tau protein has been shown to exhibit extended conformations in crowded environments to facilitate intermolecular interactions and to allow the formation of nano-clusters [66]. Separate studies have revealed that the disordered region of nucleophosmin protein and poly-uridine RNA both display extended conformations and increased dynamics on the pathway to phase separation [ 67‒ 69] . The conformational dynamics of RNA revealed by smFRET upon RNA-FUS interactions, is a molecular indicator to reveal the propensity for losing condensates fluidity [ 63, 70, 71] . WT FUS induces RNA to spontaneously fluctuate among several FRET states, whereas arginine and glycine mutants suppress the dynamics of RNA and are prone to form larger and solid-like condensates. Ubiquilin 2 increases the dynamics of RNA in the presence of FUS, which corresponds to its ability to suppress FUS stress granule formation in cells. Overall, smFRET provides direct access to the intramolecular conformational dynamics and binding dynamics between biomolecules. The molecular clues to understand the interplay between molecular dynamics and condensate formation are of great value to unravel the mechanism of liquid-to-solid transition.

### Fluorescence correlation spectroscopy

**Figure 6:**
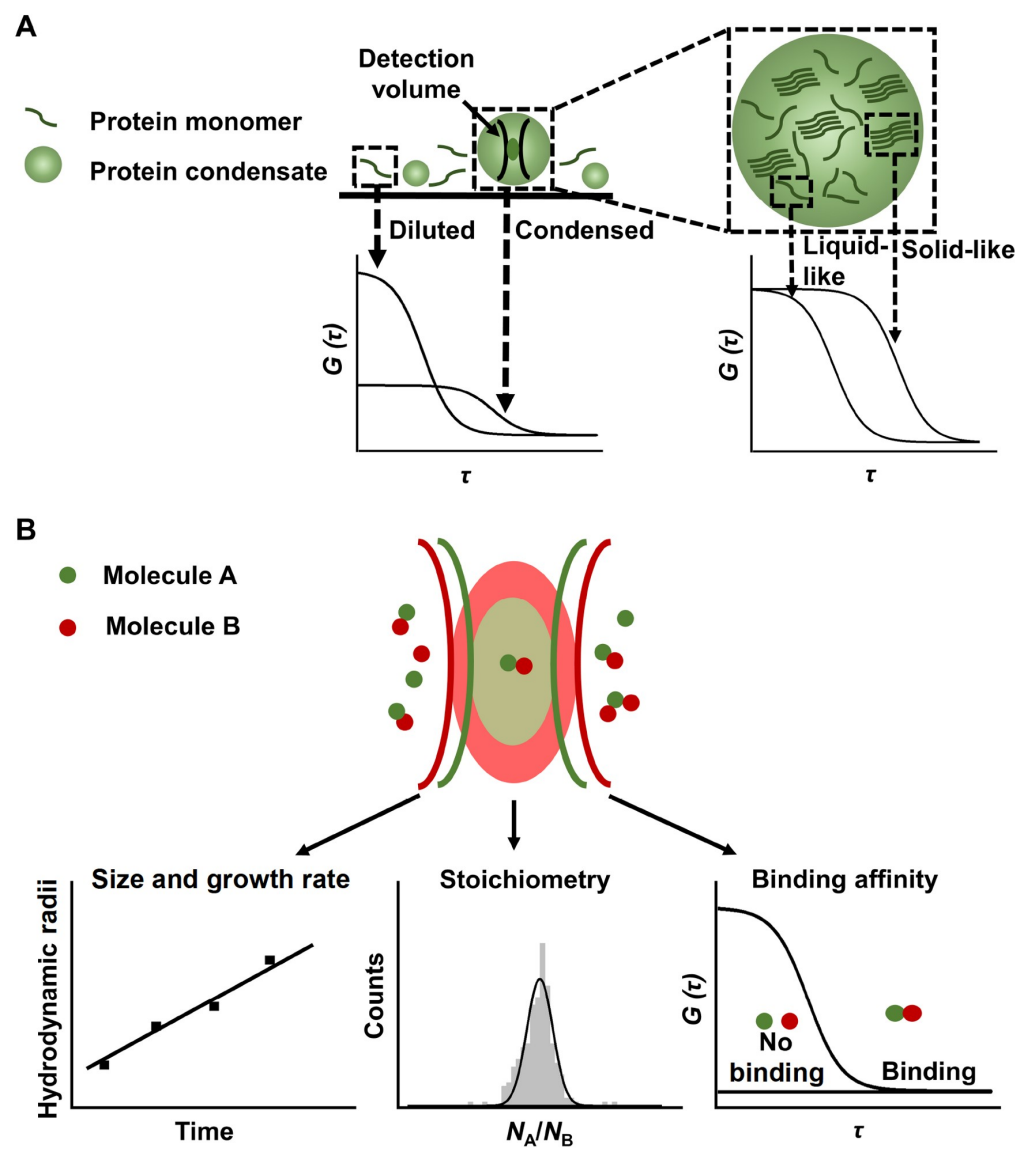
Figure 6 The schematic diagram of FCS and FCCS (A) FCS differentiates protein monomers and condensates by their different diffusion coefficients and maps the inner fluidity within condensates. (B) Size, growth rate, stoichiometry, and binding affinity of multi-component condensates at the nanoscale can be quantified by FCCS.

Fluorescence correlation spectroscopy (FCS) uses correlation analysis to extract the dynamic properties of any physical and chemical processes altering the fluorescence intensities of individual molecules. FCS measurements are performed with confocal fluorescence microscopes, which focus the excitation laser beams into diffraction-limited focal spots. Diffusion of fluorophore-labeled molecules and particles in and out of the laser focal spots leads to sudden changes in fluorescent signals, from which FCS measurements quantify the average dwell times of molecules and particles within the focal volumes to estimate their diffusion coefficients and hydrodynamic radii [72]. In addition, the number of fluorophore-labeled molecules or particles can be directly quantified ( Figure 6A).

FCS can be performed *in vitro* and in living cells. In 1998, FCS was used to detect single Aβ aggregates in the cerebrospinal fluid of Alzheimer’s disease patients [73]. Aβ aggregates in the samples from patients serve as seeds to facilitate aggregation of fluorescence-labeled Aβ, leading to high-intensity peaks and long diffusion times, whereas fluorescence-labeled Aβ in the control samples does not aggregate under experimental conditions and exhibits almost no high-intensity peak, demonstrating that FCS is capable of detecting aggregations in clinical samples. Due to the variation in the refractive index of condensate-forming systems, ultrafast-scanning FCS (usFCS) has been developed to precisely measure concentrations and diffusion coefficients in condensed and diluted phases [74]. Then, usFCS was used to characterize the full coexistence curves of LAF-1 proteins, showing that the intra-droplet concentration is surprisingly low. Diffusion coefficients of probes of different sizes within the LAF-1 condensates indicate that the effective mesh sizes of LAF-1 condensates are 3–8 nm, which is consistent with the theoretical analysis and experimental results in *C*. *elegans* embryos, suggesting that the condensates are solvent-rich and full of permeable voids to permit free diffusion of small and folded biomolecules [74]. The semi-diluted and void-rich model of LAF-1 provides a new framework for understanding the working mechanism of liquid-phase organelles. In addition, FCS measurements can reveal the heterogeneity of condensates *in vitro*, such as the DNA-PLL condensate, whose center and edge display different diffusion behaviors [75].

In living cells, FCS has been used to quantify intracellular protein concentration, which is essential because LLPS is concentration-dependent and the physiological concentration may vary among different cells. FCS is able to obtain precise concentrations within individual cells and correlates them with protein copy numbers in hubs formed from low-complexity domains [26]. Moreover, FCS provides higher spatial and temporal resolutions than the commonly used FRAP method to differentiate the heterogeneity of condensates within living cells. FUS and zona occludens proteins both display fast and slow diffusion components in live cells, corresponding to individual freely diffusing molecules and large soluble complexes, respectively [ 76, 77] . In addition, mutating the RNA binding domains of FUS decreases the fraction of slow diffusing RNA-FUS complexes, reduces the solubility of FUS, and enhances the LLPS of FUS according to FCS results [77].

Fluorescence cross-correlation spectroscopy (FCCS) is an extension of FCS that uses two overlapped focused laser beams to excite two fluorophores of different wavelengths [78]. FCCS is a highly sensitive quantitative method to detect signals from complexes and particles containing two kinds of fluorophores, with which condensates formed at the nanoscale have been visualized, and their sizes, growth rates, molecular stoichiometry, and binding affinities between molecules within condensates are quantified ( Figure 6B) [ 79, 80] . With this information, LLPS of cGAS and DNA has been examined to shed light on the molecular mechanisms of phase-separation-induced cyclic GMP-AMP synthase activation [81].

Due to the optical diffraction limit, most researchers mainly focus on micrometer-sized condensates. However, without the need for imaging, the FCS-based method enables examination of LLPS at the initial stage and confirms the existence of phase separation at the nanoscale, which makes it a promising tool to monitor the dynamic transition process from miscible individual molecules to multi-component condensates.

## Conclusions and Perspectives

A comprehensive understanding of biomolecular condensates and aggregations is crucial to understand their biological roles in living cells. Hence, it is important to have a series of technical tools to distinguish liquid- and solid-like assemblies, track the phase transition in real time, determine their structures and mechanical properties with high spatial resolution, and characterize their dynamic and thermodynamic properties at the molecular level. In this review, we summarize the working principles and examples of several commonly used single-molecule techniques, which are highly sensitive methods to capture or even manipulate individual biomolecules ( Table 1). We showcase the power of single-molecule techniques to extract important dynamic and mechanical properties, which can rarely be measured by other ensemble methods.

**Table:** **Table 1** Summary of single-molecule techniques

Techniques	Applicable conditions	Unique advantages
AFM	*In vitro*	Direct mapping of structural heterogeneity, mechanical property, and condensation.
Optical tweezers	*In vitro* and in cells	Controllable nucleation and fusion events of condensates.
DNA curtains	*In vitro*	High-throughput examination of hundreds of DNAs and proteins.
Single particle tracking	*In vitro* and in cells	Capturing molecular diffusion behaviors within and outside condensates. Examining intermolecular interactions with and without condensates.
Single-molecule counting and smFRET	*In vitro*	Capturing intramolecular conformational dynamics and intermolecular interactions with and without condensates.
FCS and FCCS	*In vitro* and in cells	Quantifying protein concentrations, diffusion coefficients, and heterogeneity within condensates. Determining the size, growth rate, and molecular composition of nanoscale condensates.

One advantage of single-molecule techniques is that they enable direct examination of condensation under close-to-physiological conditions with high spatial resolution. For example, unlike electron microscopy which needs sample staining and fixation [ 10, 22] , AFM can track the formation of condensation in real time with nanoscale resolution. SPT, FCS, and optical tweezers are all capable to examine the dynamics of molecules and condensates *in vitro* and in living cells directly. In addition, due to the low sensitivity of ensemble methods, high concentrations or over-expressed biomolecules and crowding agents, such as polyethylene glycol (PEG), are usually used to induce the formation of micrometer-sized condensates [ 20, 31] , which might not represent the real physiological conditions in living cells [82]. The high sensitivity of single-molecule techniques enables researchers to capture condensates formed at the nanoscale under physiological concentrations without the need for crowding agents. Another advantage of single-molecule techniques is to directly provide quantitative measurements of nanoscale mechanical property, molecular diffusion coefficient, binding affinity, compaction rate, nucleation size, and molecular composition, all of which are essential information to understand LLPS and cannot be easily obtained by other methods. The third advantage is that various single-molecule techniques provide a broad spectrum of information from molecular dynamics to micrometer scale morphology to thoroughly understand the interplay between individual molecules and condensates, including how modulation of molecular dynamics caused by mutations alters condensation and phase transition and how phase separation and transition from liquid- to solid-state further modulate molecular dynamics and interactions within them, leading to distinctive biological outcomes.

Although single-molecule techniques exhibit several advantages over ensemble tools, the application of single-molecule techniques in LLPS and phase transition is still in the early stage. To date, optical tweezers have only been used to examine LLPS *in vitro*, whereas they are fully capable to manipulate particles in living cells [ 83, 84] and should be applied to examine condensates in cells in the future. Moreover, DNA curtains might be extended to RNA curtains to detect RNA-involved condensation. Other advanced techniques, such as functionalized AFM tips, multi-color FRET, polarized FCS, STED-based FCS, and high-throughput magnetic tweezers, will also benefit future research [ 85– 90] . Thus, single-molecule techniques will serve as an indispensable part of toolsets to dissect molecular mechanisms of LLPS-mediated biological processes and phase transition-related diseases.
